# Cortical processes of speech illusions in the general population

**DOI:** 10.1186/s12868-016-0301-8

**Published:** 2016-10-18

**Authors:** E. Schepers, L. Bodar, J. van Os, R. Lousberg

**Affiliations:** 1 Department of Psychiatry and Psychology, Maastricht University Medical Centre, Maastricht, The Netherlands; 2 Department of Psychosis Studies, King’s College London, King’s Health Partners, Institute of Psychiatry, London, UK

**Keywords:** Speech illusion, White noise, Cortical oscillations, Reduced alpha activity

## Abstract

**Background:**

There is evidence that experimentally elicited auditory illusions in the general population index risk for psychotic symptoms. As little is known about underlying cortical mechanisms of auditory illusions, an experiment was conducted to analyze processing of auditory illusions in a general population sample. In a follow-up design with two measurement moments (baseline and 6 months), participants (n = 83) underwent the White Noise task under simultaneous recording with a 14-lead EEG. An auditory illusion was defined as hearing any speech in a sound fragment containing white noise.

**Results:**

A total number of 256 speech illusions (SI) were observed over the two measurements, with a high degree of stability of SI over time. There were 7 main effects of speech illusion on the EEG alpha band—the most significant indicating a decrease in activity at T3 (t = −4.05). Other EEG frequency bands (slow beta, fast beta, gamma, delta, theta) showed no significant associations with SI.

**Conclusion:**

SIs are characterized by reduced alpha activity in non-clinical populations. Given the association of SIs with psychosis, follow-up research is required to examine the possibility of reduced alpha activity mediating SIs in high risk and symptomatic populations.

## Background

Cognitive and neuroimaging studies (in both clinical and general populations) have focussed on the phenomenology and neurophysiological correlates of hallucinations in clinical and non-clinical populations. Theoretical accounts of the cognitive basis of hallucinations have focused on the hypothesis of a distorted balance between imagery and perception [1]. Alterations in information processing, in which the system assigns an increased influence to top-down factors (perceptual expectation through mental imagery), at the expense of bottom-up information (incoming sensory information), may contribute to the beginning of hallucinations. The hypothesized increased top-down influence of imagery on perception is supported by the finding that the hallucinatory severity correlates positively with the influence of mental imagery on auditory tone detection [2]. Functional neuroimaging has frequently been used to measure neural activity during hallucinations, as well as to assess associations with brain change [3–5]. For example, it has been reported that more intense hallucinations are associated with smaller left anterior superior temporal gyrus volumes. This suggests that the dysfunction underlying the production of auditory hallucinations affects brain regions subserving language processing [3]. Woodruff et al. [4] suggest that auditory hallucinations involve activation of brain areas associated with the perception of external speech, in addition to misperception of internal speech. In a study conducted by Shergill et al. [5], it was observed that 6–9 s before the onset of hallucinatory activity, the left inferior frontal and right middle temporal gyri were triggered. In another study, the left insula and bilateral temporal gyri were activated during the perception of a hallucination. In combination, these studies provide evidence for cortical brain changes during or even before the occurrence of hallucinations.

Unlike hallucinations, illusions are distorted interpretations of a ‘real’ external stimulus. Illusions are believed to be precursors of hallucinations in the early stages of psychosis [6]. However, illusions are more common than hallucinations and illusions do not necessarily lead to hallucinations. Hoffman and colleagues reported that speech illusions may signal an increased risk for transition to psychotic disorder in a prodromal population [7]. Galdos and colleagues described an experimental speech illusion task, designed to prime and elicit auditory illusions. In this task, participants are presented with three different categories of sound fragments: fragments containing ‘pure’ white noise; fragments of white noise mixed with a barely audible speech in the background and fragments with white noise mixed with clearly audible speech. After hearing fragments, participants (controls, patients with psychotic disorder and their siblings) were asked to indicate whether they heard a sentence with positive, negative or neutral content, or heard a voice but were uncertain whether the content was positive, negative or neutral, or heard no voice at all. A speech illusion (SI) was defined as a white noise fragment in which any speech was heard. In the control group, there were 9 % speech illusions, in the group of patients with psychotic disorder this was 30 %. Therefore, although the rate of speech illusions in the patient group was much higher than in control participants, speech illusions were also relatively common in the non-psychotic population. In addition, siblings of patients had higher rates of speech illusions (14 %) than controls, suggesting speech illusions index risk for psychosis [8].

Studies investigating the role of cortical mechanisms of illusory perceptions are primarily conducted in the visual domain [9–12], and to a lesser extent in the auditory domain. According to recent literature, the power of alpha oscillations is associated with perception and cognitive processes [13, 14]. Romei et al. [13] suggested that the activity of alpha oscillations in the visual cortex mediates whether stimuli are perceived or not, showing that low alpha power (high excitability) may facilitate visual perception and high alpha power may inhibit perception of stimuli. Comparable effects are reported in studies investigating the cortical oscillations during auditory perception. Weisz et al. [15] studied the role of alpha oscillations in tinnitus. They found a significant decrease in the power of ongoing alpha activity for the tinnitus group compared to normal hearing controls. The reduction was predominantly found in the bilateral temporal regions of the auditory cortex. Similar findings of an association between alpha activity and auditory perceptions were reported in a study of Müller et al. [16]. These authors recorded brain activity while participants listened to familiar as well as unknown music that was partly replaced by sections of noise. During the noise fragments, participants reported a stronger illusory music perception for familiar songs as compared to unfamiliar songs. Leske et al. [17] investigated oscillatory activity during a Zwicker Tone illusion with different notch widths. In this paradigm, a notch-filtered auditory noise induces an ‘auditory afterimage’. Participants reported a stronger auditory perception with increasing notch widths. The increasing notch widths were associated with decreased alpha power, showing an inverse relation between ‘auditory afterimage’ and alpha power. Reported associations between altered alpha activity and illusionary phenomena in these studies suggest it may be productive to examine the hypothesis of alterations in oscillatory activity during a pure white noise fragment reported as a speech illusion.

We wished to examine the auditory speech illusion experiment, reported by Galdos et al. [8], and replicated by Catalan et al. [18], using a repeated measure paradigm (baseline and 6 months) in a general population sample. The main objective was to demonstrate that cortical processing of a speech illusion (SI) would differ from a correctly judged white noise fragment. Based on the findings of previous research, our main hypothesis focussed on changes on the alpha band. However, we planned to analyse the complete frequency spectrum, ranging from delta to gamma. Age and sex impact EEG activity, and were included as confounding variables in the statistical models.

## Methods

### Participants

Eighty-three persons participated, of which 66 took part both at baseline and at follow-up. Participants were recruited from the population of Maastricht (population: 120,000) using flyers at public places. Exclusion criteria were the use of antipsychotic, anti-epileptic, antidepressant or anxiolytic medications during the past year or structural use of more than 5 units of alcohol per day. Participants were asked to avoid the use of alcohol the day before the experimental session. They were also asked not to use caffeine-containing beverages 3 h before the experiment. Participants with hearing problems were excluded. Participants reported on the presence of a first- or second-degree relative diagnosed with a psychotic disorder. Compensation for participating in the two measurements was 50 €.

### Ethics statement

The study was conducted according to the principles of the Declaration of Helsinki and the medical ethics committee at Maastricht University Medical Centre approved the study. Participants gave written informed consent before the start of the experiment.

### Design and procedure

The study design included two measurements, at baseline and at 6 months. On both occasions, the white noise task was administered in a psychophysiological laboratory.

Each participant was exposed to 75 sound fragments, equally divided over three subgroups: 25 fragments containing ‘pure’ white noise; 25 fragments of white noise mixed with barely audible speech in the background; 25 fragments with white noise mixed with a clearly audible speech. The affective context of the sentence of the clearly audible speech fragments was either positive, negative or neutral. For example: ‘Sport is good for health’, ‘I think it is going to rain today’ or ‘Madrid is the capital of Spain’. Each fragment had a duration of 4.3 s; the spoken sentence was constructed to last as close as possible to 4.3 s. The 75 fragments were presented in random order. After each fragment, participants were asked to press a button on a keyboard (just in front of them) to characterize the fragment: 1: endorsed hearing speech with positive content, 2: endorsed hearing speech with negative content, 3: endorsed hearing speech with neutral content, 4: no speech heard, and 5: endorsed hearing speech but uncertain whether it was positive, negative or neutral. As stated above, a speech illusion was defined as a white noise fragment in which any speech was heard (thus either option 1, 2, 3 or 5). The sound fragments were binaurally presented through headphones (Plantronics) and making use of a Soundmax integrated digital HD audiodriver sound card. The protocol was guided by the software package ‘Presentation’ (Version 13.0, Neurobehavioral Systems, Inc.). Markers were placed in a separate EEG marker-channel to indicate the start and the end of a fragment as well as at the moment the participant pressed the ‘answer-button’. During the fragment, the word “listen!” was displayed on the computer screen (placed in front of the participant). Immediately after a fragment, the five answer options were shown and participants were asked to rate the fragment. A new fragment was started 1 s after an answer was given. Response time was calculated as the time (measured in ms) between the end of a fragment and the button push. The total duration of the task was variable, since the response times varied per participant. The average task duration was 8.8 min (SD = 0.88, range: 7.3–11.9).

### EEG measurement

Ag/AgCl electrodes were attached to the participant’s head at the following locations: Fz, F3, F4, Cz, C3, C4, Pz, P3, P4, T3, T4, Oz, O1 and O2, using the international 10–20 system [19]. Electro-oculogram electrodes were placed 1 cm under the midline of both eyes to measure ocular activity. A reference electrode was placed on each ear lobe. The two reference electrodes were linked to each other. A ground electrode was placed at the forehead. To reduce resistance, Nuprep scub gel was used. Conductive gel (Ten20 conductive) was used to fill the electrodes. Impedances were kept below 5 kΩ. BrainAmp Research Amplifier (Brain Products, resolution 0.1 μV) was used for all recordings. EEG was sampled with 1000 Hz.

### EEG offline data processing

Offline data processing was performed with BrainVision Analyser 2.0 (Brain Products, München, Germany). In a first step, data was offline filtered (band pass 0.5–50 Hz). The data were divided into 4096 ms segments, marked by the onset and end of the fragment. For each segment, a FFT transformation was applied after which the frequency bands delta (1–3 Hz), theta (3–7.5 Hz), alpha (7.5–13 Hz), slow beta (13–20 Hz), fast beta (20–30 Hz) and gamma (30–48 Hz) were computed. Because of skewed non-normal distributions of the EEG band data, a log-transformation on each EEG band was carried out. The log-transformed EEG variables were normally distributed.

### Statistical analysis

Given the hierarchical structure of the EEG dataset, consisting of 25 white noise fragments (level 1) clustered within individuals (level 2), clustered within two experimental sessions (level 3), multilevel random regression analyses were performed [20]. In order to test which covariance structure yielded the best fit, various covariance structures were tested. The covariance structure which best fitted the data was an autoregressive (AR1) structure. All models were tested with a random intercept and random slope for the number of fragments. The EEG frequency bands of each location were the dependent variables. The following variables served as covariates: EOG (left and right activity), age (in years) and sex (male = 0, female = 1). A dummy variable ‘speech illusion’ (SI: yes or no) was used as the independent variable of main interest. In addition, the independent variables response time, fragment number, 1/fragment number (as a non-linear fragment effect) and measurement were incorporated in the model. All statistical analyses were performed with SPSS version 21.0. *p* values ≤0.05 were considered as statistically significant.

## Results

### Participant characteristics

Eighty-three (Age: µ = 37.2 (SD = 17.8); sex: 30 men, 53 women) participated at baseline, sixty-six (Age: µ = 38.1 (SD = 17.9); sex: 26 men, 40 women) were seen at 6-month follow-up. Analyses were performed to check whether there was selective attrition, which revealed no large or significant differences with regard to rate of SI (*p* = 0.26), age (*p* = 0.38) and sex (*p* = 0.23). Two participants indicated they had a relative with a psychotic disorder, 4 participants did not know and the remainder did not have a relative with a psychotic disorder.

### Rate of speech illusions

Each participant listened to 75 fragments per measurement occasion, of which 25 were pure white noise fragments. Thus, a maximum of 25 SIs could occur per measurement occasion. At baseline, 83 participants generated a total of 148 SIs (7.1 % = 148 SI/[83 participants * 25 noise fragments]). The total number of SIs made by the n = 66 participants at follow-up was 108 or 6.5 %.

The number of SIs was divided into three categories: 3 or more during a measurement occasion, 1–2 during a measurement occasion and zero SIs. The frequency of these three categories over the two measurements can be observed in Table 1. The Chi square test of this table was significant (χ^2^ = 20.5; df = 4; *p* < 0.001), indicating relative stability over the two sessions. Twenty participants consistently had no SI at baseline and follow-up, 9 persons generated 3 or more SIs at both measurements. In the latter group, no one had a family member with a psychotic disorder.

**Table 1:** Rate of SI (number of participants) over both measurements

	Measurement 2			
	SI = 0	1 ≤ SI ≤ 2	SI ≥ 3	Total
*Measurement 1*				
SI = 0	20	9	4	33
1 ≤ SI ≤ 2	11	7	2	20
SI ≥ 3	2	2	9	13
Total	33	18	15	66

### Response time

**Fig. 1:**
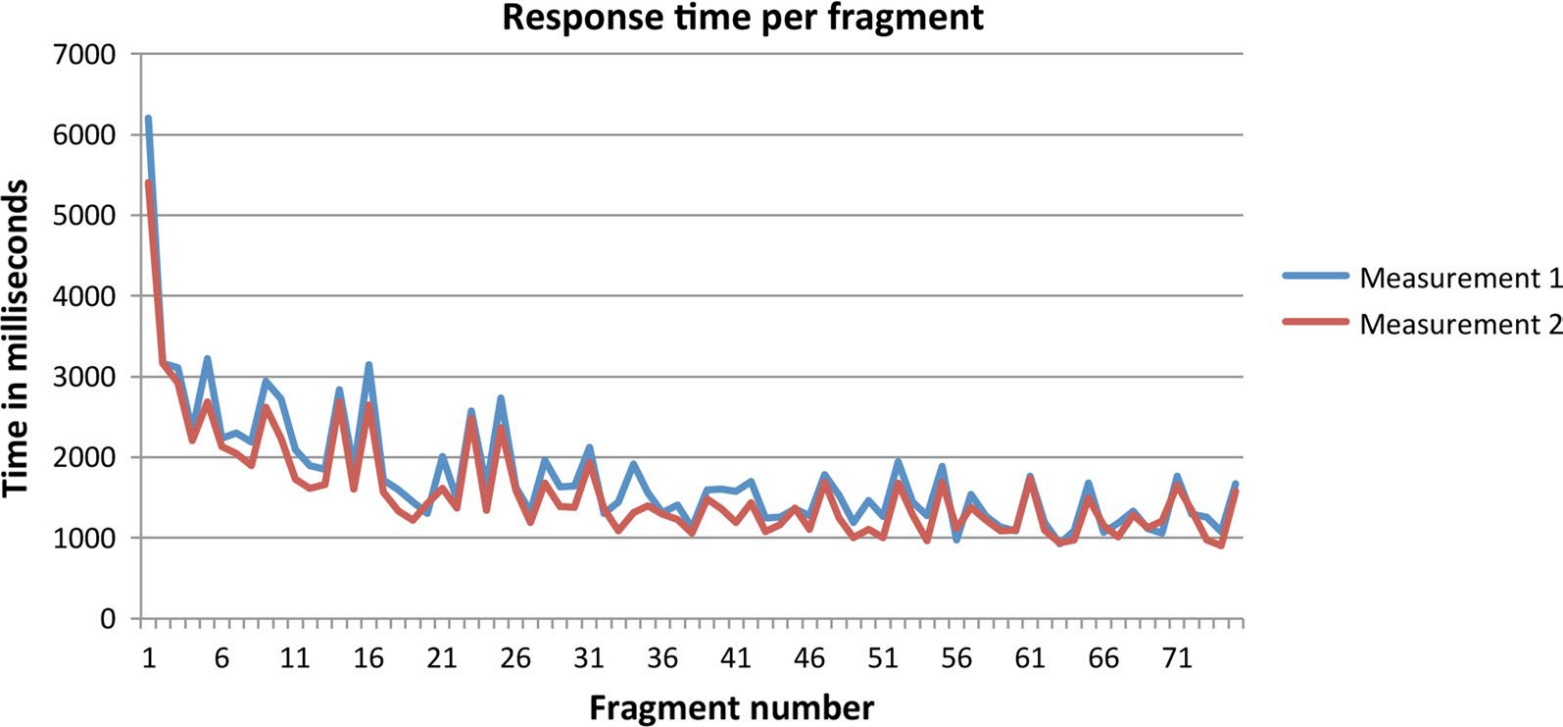
Response time per fragment

Although no instruction was given to judge the fragments as quickly as possible, analysis of the response time is important in order to better understand the (cortical) decision process underlying SIs. The response time was defined as the time between the end of the stimulus and the push on the button. An inspection of the frequency distribution of the response times (the dependent variable) showed that there were some extreme (outlying) values. It was decided to remove all response times above 10000 ms (0.6 % of the data). Figure 1 gives an illustration of how response times decrease during the series of 75 fragments. The curve is indicative for a non-linear (inverse) decreasing effect, the largest decrease being observed during the first twenty fragments. Both the linear effect as well as the inverse (1/fragment) effect was associated with the response time (*p*’s < 0.001). In addition, it can be observed that the mean response time of the fragments at baseline (1745 ms) was longer compared to follow-up (1563 ms; *p* < 0.001). Further analyses revealed that increasing age was accompanied by longer response times (*p* = 0.001). SI was not associated with response time (*p* = 0.78).

### EEG during SI

**Fig. 2:**
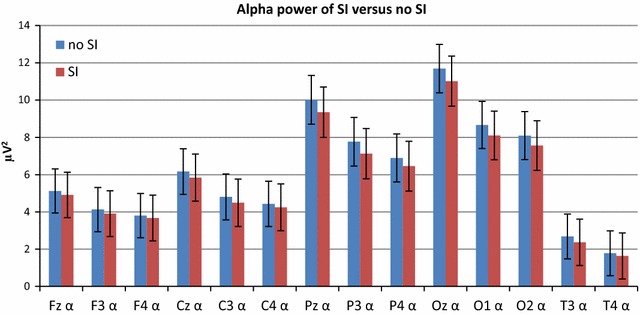
Alpha power of SI versus no SI

The recorded EEG of one participant did not fulfil the resistance criterion (all electrodes >5 kΩ). Consequently, this person was excluded from the EEG analyses. In Fig. 2 the raw, untransformed alpha power of SI versus no SI are displayed for all 14 locations. At all locations, alpha power during a speech illusion was reduced. Significant main effects of SI were observed at locations C3, Pz, P3, O1, O2, T3 and T4. Correcting for the multiple testing (applying the Bonferroni criterion), *p* values less than 0.0035 (0.05/14) were considered significant. This is conservative, given that Bonferroni correction may result in diminished power to detect differentiation among pairs of sample collections [21]. After applying Bonferroni correction, 4 cortical locations: C3, P3, T3 and T4 remained significant. Table 2 shows the log transformed alpha band power with the corresponding T-values at all electrodes. The most significant main effect was at T3 alpha (T = −4.05). Finally, a series of interaction models was run to test whether there were speech illusion * measurement interaction effects. No significant interaction effects were found. At all other EEG frequency bands no significant main and interaction (SI*measurement) effects were apparent.

**Table 2:** Estimates of the log power of the alpha band during SI versus no SI with corresponding t-and *p* values

	Estimate no SI	Estimate SI	t-value	*p* value
Log Fz α	0.710	0.691	−1.359	0.174
Log F3 α	0.616	0.592	−1.696	0.090
Log F4 α	0.580	0.565	−1.063	0.288
Log Cz α	0.790	0.766	−1.865	0.062
Log C3 α	0.681	0.652	−*2.120*	*0.034*
Log C4 α	0.646	0.628	−1.349	0.177
Log Pz α	1.001	0.971	−*1.972*	*0.049*
Log P3 α	0.890	0.853	−*2.542*	*0.011*
Log P4 α	0.838	0.810	−1.907	0.057
Log Oz α	1.068	1.042	−1.879	0.060
Log O1 α	0.938	0.909	−*2.074*	*0.038*
Log O2 α	0.908	0.878	−*2.075*	*0.038*
Log T3 α	0.428	0.374	−*4.045*	*<0.001*
Log T4 α	0.250	0.214	−*2.670*	*0.008*

Italic numbers indicate significant locations (*p* < 0.05)

## Discussion

### Protocol validation

In this study, an attempt was made to link speech illusions to cortical activity in a general population sample using a specific protocol. The results of the analyses of the response time can be regarded as a validation of the protocol, as both the decreasing non-linear response times over fragments as well as the mean decreased response time in measurement 2 have face validity: participants learned to perform the task faster and participated twice in the same experiment. Also, the finding that older people displayed longer response times is in line with expectation.

At baseline, there were 13 participants who reported three or more SIs. Six months later, almost 70 % (9 out of 13) again displayed three or more SIs. This observation suggests substantial stability of SI over time. In addition, no significant EEG activity * measurement interactions were observed for any dependent variable.

### Main findings

The main finding of this experiment was that speech illusions can be characterized by significant changes in cortical activity in the EEG alpha band across different cortical locations.

Seven SI main effects were observed on the alpha band, the most significant at temporal locations. The decrease of alpha oscillations during an auditory illusion supports findings of earlier studies. The perception of a sound is associated with decreased alpha activity, indicating high excitability. As described earlier, the most significant reduction in alpha oscillations was found at the temporal region, a result also reported by Müller and Weisz. Higher excitably of the temporal lobes is conform expectation given that the auditory cortex is located within the temporal lobe, and the same area is involved in hallucinations. As mentioned in the introduction, research has shown that activity in the left temporal lobe is altered during hallucinatory states [5]. More specifically, just before a hallucination, the left inferior frontal and right middle temporal gyri are recruited, and during the (hallucinatory) perception, the left insula and bilateral temporal gyri are activated [5]. Significantly reduced alpha activity, however, was not only observed at the temporal regions. It is possible that other regions than the temporal regions are also involved in alpha reduction during an illusion.

The fact that SIs were reported in a sample of the general population without a psychotic disorder supports the idea of phenomenological continuity of psychosis across clinical and non-ill populations [22]. Phenomenological continuity relates to the notion that psychotic experiences can occur outside psychotic disorder and reflect the psychometric liability to psychosis as distributed in the population [22].

### Methodological issues

A number of participants were lost to follow-up, i.e. did not participate in follow-up measurement at 6 months. However, analyses suggested little potential for differential attrition. A second limitation is that from a 14-leads EEG no inferences can be made on which cortical sources are active. Future studies may combine EEG measurements with fMRI in order to pinpoint source location.

Future efforts may focus on cortical processing of SI in healthy controls as compared to patients with a psychotic disorder. Two opposing hypotheses may be tested. On the one hand, a stronger and more pronounced cortical activity underlying SIs may be expected in patients; on the other hand, involvement of other cortical mechanisms may differentiate patients from controls. In addition, the cortical relationship between illusions and hallucinations may be explored in a prodromal population in order to understand the process of transition to psychotic disorder as reflected by cortical activity underlying psychotic symptoms.

## Conclusions

Speech illusions are characterized by reduced alpha activity across different cortical locations in non-clinical populations. Given the association of SIs with psychosis, follow-up research is needed to examine the possibility of reduced alpha activity mediating SIs in high risk and symptomatic populations.

## Abbreviation

SI: speech illusion
